# Multiregional blood-brain barrier phenotyping identifies the prefrontal cortex as the most vulnerable region to ageing in mice

**DOI:** 10.1093/braincomms/fcaf332

**Published:** 2025-09-10

**Authors:** Isabel Bravo-Ferrer, Katrine Gaasdal-Bech, Chiara Colvin, Hollie J Vaughan, Jonathan Moss, Anna Williams, Blanca Díaz Castro

**Affiliations:** Centre for Discovery Brain Sciences, The University of Edinburgh, Edinburgh EH16 4SB, UK; UK Dementia Research Institute at the University of Edinburgh, Edinburgh EH16 4SB, UK; Centre for Discovery Brain Sciences, The University of Edinburgh, Edinburgh EH16 4SB, UK; UK Dementia Research Institute at the University of Edinburgh, Edinburgh EH16 4SB, UK; Institute for Neuroscience and Cardiovascular Research, The University of Edinburgh, Edinburgh EH16 4SB, UK; British Heart Foundation and UK Dementia Research Institute Centre for Vascular Dementia Research, Edinburgh EH16 4SB, UK; Centre for Discovery Brain Sciences, The University of Edinburgh, Edinburgh EH16 4SB, UK; UK Dementia Research Institute at the University of Edinburgh, Edinburgh EH16 4SB, UK; Institute for Neuroscience and Cardiovascular Research, The University of Edinburgh, Edinburgh EH16 4SB, UK; British Heart Foundation and UK Dementia Research Institute Centre for Vascular Dementia Research, Edinburgh EH16 4SB, UK; Centre for Discovery Brain Sciences, The University of Edinburgh, Edinburgh EH16 4SB, UK; UK Dementia Research Institute at the University of Edinburgh, Edinburgh EH16 4SB, UK; Centre for Regenerative Medicine, Institute for Regeneration and Repair, Edinburgh Bioquarter, University of Edinburgh, Edinburgh EH16 4UU, UK; UK Dementia Research Institute at the University of Edinburgh, Edinburgh EH16 4SB, UK; Centre for Regenerative Medicine, Institute for Regeneration and Repair, Edinburgh Bioquarter, University of Edinburgh, Edinburgh EH16 4UU, UK; Centre for Discovery Brain Sciences, The University of Edinburgh, Edinburgh EH16 4SB, UK; UK Dementia Research Institute at the University of Edinburgh, Edinburgh EH16 4SB, UK; Institute for Neuroscience and Cardiovascular Research, The University of Edinburgh, Edinburgh EH16 4SB, UK; British Heart Foundation and UK Dementia Research Institute Centre for Vascular Dementia Research, Edinburgh EH16 4SB, UK

**Keywords:** blood-brain barrier, ageing, astrocyte endfoot, brain endothelial cell, transmission electron microscopy

## Abstract

Age-associated vascular alterations make the brain more vulnerable to neuropathologies. Research in humans and rodents has demonstrated structural, molecular, and functional alterations of the aged brain vasculature that suggest blood-brain barrier dysfunction. However, these studies focused on particular features of the blood-brain barrier and specific brain regions. Thus, it remains unclear if and which blood-brain barrier age-associated phenotypes are conserved across brain areas. Moreover, there is very limited information about how blood-brain barrier dysfunction and cell-specific phenotypes relate to each other. In this manuscript, we use immunofluorescence, transmission electron microscopy, and permeability assays to assess how age-associated blood-brain barrier molecular, structural, and functional phenotypes correlate between the blood-brain barrier cell types at three brain regions (prefrontal cortex, hippocampus, and corpus callosum) during mouse early ageing. We discovered that at 18–20 months of age, changes to the mouse blood-brain barrier are subtle. The prefrontal cortex blood-brain barrier is the most affected by age, with alterations in brain endothelial cell protein expression, blood-brain barrier permeability, basement membrane thickness, and astrocyte endfoot size when compared with young mice. Here, we deliver a detailed multicellular characterization of region-dependent blood-brain barrier changes at early stages of ageing. Our data paves the way for future studies to investigate how region-specific blood-brain barrier dysfunction may contribute to disease-associated regional vulnerability.

## Teaser

Bravo-Ferrer, Gaadal-Bech *et al*. examined age-associated alterations of the mouse blood-brain barrier across three brain regions. Using immunofluorescence, electron microscopy, and permeability assays, they identified the prefrontal cortex as the most susceptible to age (18–20 months). Their findings highlight region- and cell-specific blood-brain barrier phenotypes emerging during early ageing.

## Graphical Abstract

**Graphical Abstract:**
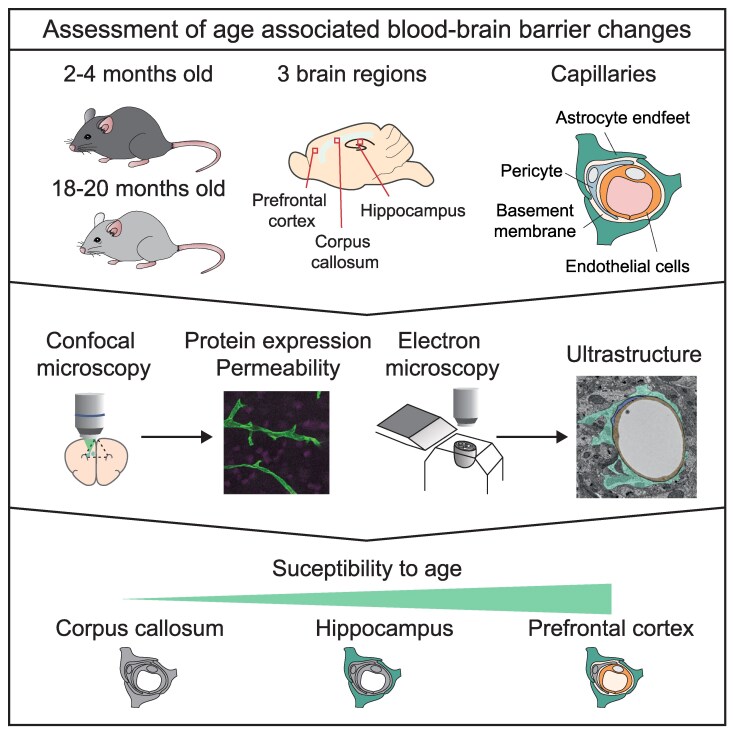

## Introduction

Age-associated alterations make the brain more vulnerable to neuropathologies.^1^ In the brain, vascular dysfunction appears as a prominent feature of age that often precedes cognitive decline.^2-4^ Research in humans and rodents has demonstrated structural,^5-7^ molecular,^8-10^ and functional^11-14^ alterations of the brain vasculature with age that suggest blood-brain barrier (BBB) dysfunction.

The BBB is essential to maintain a healthy brain microenvironment as it strictly regulates the exchange of molecules between the blood and the brain. In the capillaries, constituting 85% of the brain vasculature, the BBB is formed by the brain endothelial cells (BECs), pericytes, basement membrane, and astrocyte endfeet, which are specialized perivascular subcellular compartments. The BECs form the vessel wall and are the first barrier of the BBB. They have a luminal side facing the blood and an abluminal side that is surrounded by the basement membrane and faces pericytes and astrocyte endfeet.^15^ To form an effective BBB, BECs restrict entry by expressing robust molecular complexes called tight junctions, which firmly bind BECs, and by maintaining a low transcytosis rate, limiting the movement of non-regulated cargo.^16^ The import of necessary substances to the brain side is ensured by molecule-specific transmembrane transporters.^17^ Embracing the BECs, pericytes enhance their BBB properties and regulate blood flow.^18^ Lastly, astrocytes uniquely enwrap the brain vasculature while simultaneously contact brain cells to coordinate vascular and neural functions.^18-21^ As such, astrocytes take up nutrients transported in the blood and metabolize them,^22,23^ modulate local blood flow,^24-27^ and enable the clearance of brain by-products and toxins.^28,29^

With age, the BBB deteriorates in both mouse and humans. In BECs, the expression of tight junctions has been shown to be reduced,^5,7,8^ transcytosis increased,^12^ and vesicular and molecule-specific transporters altered.^5,10^ In addition, a reduction of pericyte vessel coverage has been reported in aged brains,^12,30,31^ while the basement membrane, the vascular extracellular matrix, increases in size.^5-7,9^ Astrocyte endfeet have also been reported to be affected by age, with alterations in the cellular polarization of the astrocyte water channel aquaporin-4 (AQP-4) in mouse and human^11,32^ and hypertrophic appearance in the brains of aged rats.^5^

Overall, numerous studies have demonstrated that age affects the BBB function, structure, and molecular profiles in both rodents and humans. However, these studies focused on particular aspects of the BBB and specific brain regions. Thus, important questions emerge. Are BBB age-associated phenotypes conserved across brain regions or are they region-specific? Is there a region-dependent relationship between changes observed in each component of the BBB?

In this manuscript, we use immunofluorescence, transmission electron microscopy (TEM), and cadaverine permeability assays to assess how age-associated BBB molecular, structural, and functional phenotypes correlate between cell types and brain regions during mouse ageing. We pay particular attention to the BECs, which form the main barrier, and the astrocyte endfeet, highly understudied in this context, despite their essential roles as mediators of vascular and neural functions. Moreover, we make side-by-side comparisons of three brain regions of high relevance to neurological conditions that lead to cognitive decline—prefrontal cortex, hippocampus, and corpus callosum, in both male and female mice. Our data paves the way for future studies to investigate how BBB dysfunction may contribute to disease-associated regional vulnerability.

## Materials and methods

### Mice

Animal experiments were conducted in accordance with the national and institutional guidelines [(Scientific Procedures Act) 1986 (UK), and the Council Directive 2010/63EU of the European Parliament and the Council of 22 September 2010 on the protection of animals used for scientific purposes] and had a full Home Office ethical approval. Mice were housed with no restrictions to food and water in a 12-h light/dark cycle.

All experiments were performed using C57Bl/6J mice (Charles River Laboratories, UK) of 2–3 months or 18–20 months old mice of both sexes. Mice were randomly located in the animal room, but separated in different cages based on age and sex.

### BBB permeability assay, vasculature tracing, and drug administration

Alexa 555 conjugated cadaverine (Invitrogen Cat#A30677) was dissolved in sterile PBS at 1 mg/mL. Mice received an intravenous (i.v.) injection of 7 mg/kg 2 h before perfusion. To trace the vasculature, mice received an i.v. injection 5 min before perfusion of fluorescein conjugated lectin from Lycopersicon esculentum (Vector Cat#FL-1171) at a concentration of 5 mg/kg.

### Immunofluorescence

For immunofluorescence studies, mice were subjected to terminal anaesthesia and transcardial perfusion with PBS containing 10 units/mL of heparin followed by 10% of formalin. 40 μm coronal sections were prepared using a crysotat (Leica) and kept in 0.05 M PBS, 250 mM sucrose, 7 mM MgCl_2_ and 50% glycerol at −20°C. For goat anti-CD13 and mouse anti-claudin-5 staining, antigen retrieval was performed prior to immunostaining, by incubating the tissue in 0.01 M sodium citrate buffer (pH 6) at 80°C for 30 min. To avoid lipofuscin autofluorescence sections were pre-treated with TrueBlack (Biotium, Cat# 23007) 1× in 70% ethanol for 1 min before staining. Sections were then washed three times in PBS for 10 min and incubated at room temperature for 2 h in blocking solution containing 10% of normal goat or donkey serum in PBS with 0.2 Triton X-100 with agitation. Sections were subsequently incubated in primary antibodies diluted in the blocking solution. The following primary antibodies were used: rabbit anti-NeuN (1:2000, Cell Signalling Cat# 12943S), goat anti-CD13 (1:100, R&D Cat# AF2335), mouse anti-claudin-5 (1:500; Invitrogen Cat# 35–2500 and Invitrogen Cat# 352588), rabbit anti-caveolin-1 (1:1000, Cell Signalling Cat# 3267), rabbit anti-Pecam-1 (1:1000, BD Bioscience Cat# 550274), rabbit anti-aquaporin4 (1:1000, Millipore Cat# ab3594), mouse anti-dystrophin (1:500, DSHB, RRID: AB_2618143), and mouse anti-dystroglycan (1:1000, DSHB, RRID: AB_2618140). The next day the sections were washed three times in PBS for 10 min each and then incubated with the secondary antibody diluted in blocking solution. The following secondary antibodies were used: goat anti-mouse IgG Alexa 647 (1:1000, Invitrogen Cat# A21235), donkey anti-goat IgG Alexa Plus 647 (1:1000, Invitrogen Cat# A32849), Tomato Lectin 488 (1:200, Vector Laboratories Cat# FL-1171), goat anti-rabbit IgG Alexa 647 (1:1000, Invitrogen Cat# A21244), goat anti-rat IgG Alexa 647 (1:1000, Invitrogen Cat# A21247), goat anti-mouse IgG Alexa 488 (1:1000, Invitrogen Cat# A11001), goat anti-rabbit IgG Alexa 488 (1:1000, Invitrogen Cat# A11008), donkey anti-mouse IgG Alexa 488 (1:1000, Invitrogen Cat# A21202), and donkey anti-goat IgG (1:1000, Invitrogen Cat# A21447). The sections were mounted on microscope slides in ProLong Gold antifade reagent.

### Confocal microscopy and image analysis

Fluorescent images were taken on a Zeiss LSM900 confocal laser-scanning microscope. For BBB permeability assessment, 3–5 images were taken in each region using a 25× 0.8 NA (Plan-Apochromat, Zeiss Cat# 420852-9871-000). For BEC, pericyte, and endfoot markers assessment, 3 or 4 capillaries (diameter < 10 μm) were randomly selected in each region. To do this, a rectangle area within each brain region was selected and points were randomly generated. The closest capillary to each point was then imaged using a 63× 1.4 NA oil immersion objective (Plan-Apochromat, Zeiss Cat# 420782-9900-799). For CD13, claudin-5, caveolin-1, pecam-1, dystrophin, and β-dystroglycan, images containing the whole capillary in the *z*-plane were taken. For AQP-4 images, stacks of 6 μm were taken in vessels where the lumen was clearly visible. Imaging settings remained unchanged within groups of the same experiment.

Image analysis was conducted using FIJI (ImageJ). For BBB permeability assessment, the cadaverine mean intensity in each image was measured. For the assessment of CD13, claudin-5, caveolin-1, pecam-1, dystrophin, and β-dystroglycan expression, the maximum intensity of *z*-stack projections from the images was created and channels separated. Using the lectin channel, the vessel area was delimited creating an area of interest (ROI) that was used to measure the percentage area or mean intensity within the capillary area for each marker. For AQP-4 expression assessment, a linear ROI of 5 μm was created perpendicular to the vessel containing both vascular and parenchymal AQP-4 signal. The intensity profile of the line was then plotted, and the area under the curve for both vascular and parenchymal was calculated.

### Transmission electron microscopy

Mice were first perfused with PBS, followed by a fixative solution composed of 4% paraformaldehyde (w/v) and 2% glutaraldehyde (v/v) in 0.1 M phosphate buffer. Brains were then extracted, immersed in the same fixative for 24 h at 4°C, and sliced in ice-cold PBS into 50 μm sections using a vibratome.

The tissue sections were subsequently treated with 1% osmium tetroxide in 0.1 M phosphate buffer for 30 min, followed by a dehydration process through an ascending ethanol series (50%, 70%, 95%, and 100%) and acetone. Once dehydrated, the sections were immersed into resin and left to set overnight at room temperature. Afterwards, the sections were mounted onto microscope slides, covered with coverslips, and cured at 65°C over 3 days.

ROIs including the corpus callosum, prefrontal cortex, and hippocampus were dissected from the slides, mounted onto plastic blocks, and further sectioned to ultrathin 60 nm sections for electron microscopy. These sections were then contrasted with lead citrate and uranyl acetate and imaged using a JOEL TEM-1400 Plus electron microscope at the Edinburgh Discovery Research Platform for Hidden Cell Biology. Imaging magnifications were chosen to include each vessel along with the adjacent perivascular area for analysis. All vessels analysed were capillaries (diameter < 10 μm).

### TEM analyses

The number of vessels analysed per region is listed in Table 1. Tight junction tortuosity was calculated as the ratio of the tight junction length to the diagonal of the rectangle containing the entire junction.^33^ Normalized brain endothelial cell area was calculated as the brain endothelial cell area divided by its luminal perimeter, while normalized pericyte area was obtained by dividing the pericyte area by the abluminal perimeter of the brain endothelial cell. Similarly, normalized endfoot area was measured as the ratio of the endfoot area to the perimeter of its basement membrane. Endfoot and pericyte coverage was quantified as the length of the endfoot or pericyte basement membrane normalized to the brain endothelial cell abluminal perimeter and expressed as a percentage. To quantify endfoot number, we considered individual endfeet as the astrocyte processes in contact with the basement membrane that were delineated by distinct plasma membrane boundaries. Finally, endfoot mitochondria area was analysed measuring the total area occupied by mitochondria within the endfoot and endfoot mitochondria density was expressed as a fraction of the total endfoot area (Table 2).

**Table 1:** Condition distribution of the images used for the TEM study

Number of		Prefrontal cortex	Hippocampus	Corpus callosum
Vessels	Young (female/male)	30 (15, 15)	30 (15, 15)	29 (14, 15)
	Older (females/male)	30 (15, 15)	25 (10, 15)	29 (15, 14)
Mice	Young (female/male)	6 (3, 3)	6 (3, 3)	6 (3, 3)
	Older (females/male)	6 (3, 3)	5 (2, 3)	6 (3, 3)

**Table 2:** Summary of measurements taken for the analysis of TEM images

Parameter	Measurement
Tight junction tortuosity	Length of the tight junction/diagonal of the rectangle containing the complete tight junction^34^
Normalized brain endothelial cell area	Brain endothelial cell area/brain endothelial cell luminal perimeter
Pericyte basement membrane thickness	Pericyte basement membrane area/Pericyte basement membrane length
Endfoot basement membrane thickness	Endfoot basement membrane area/endfoot basement membrane length
Endfoot mitochondria density	Endfoot mitochondria area/endfoot area
Pericyte coverage	Length of the pericyte basement membrane × 100/brain endothelial cell abluminal perimeter
Endfoot coverage	Length of the endfoot basement membrane × 100/brain endothelial cell abluminal perimeter
Normalized pericyte area	Pericyte area/brain endothelial cell abluminal perimeter
Normalized endfoot area	Endfoot area/endfoot basement membrane perimeter

### Statistical analysis

All analyses were performed blinded to the conditions of study. Data were analysed using GraphPad Prism or R. Number of animals per experiment was determined based on previous work performing similar measures. All comparisons contained more than one datapoint per animal and no exclusions were made. Thus, to be able to consider multiple measures per animal to take within-animal variability into consideration without introducing pseudo-replication, we used linear mixed-effect modelling (LMM) for our analyses (https://github.com/Spires-Jones-Lab/Linear-mixed-models-R). For all figures except Supplementary Figs. 1B–D and 7, we considered age as the only fixed effect—LMM; Measurement ∼ Age + (1 | Animal) + (1 | Sex). For Supplementary Figs. 1B–D and 7, we considered both age and sex as fixed effects—LMM; Measurement ∼ Age × Sex + (1|Animal). For the EM studies, female and male samples were processed separately, thus, sex and batch could not be statistically differentiated. In either case, after assembling an initial LMM, the normality of the residuals was assessed using Shapiro-Wilk test. To meet model assumptions, data with non-normal residuals were transformed using Tukey Ladder of Powers. To identify significance main effects, Type 3 analysis of variance (ANOVA) with Satterthwaite approximation (to estimate effective degrees of freedom by accounting for uncertainty in the variance estimates from the random effects) was run on the LMM. Graph represent all datapoints measured with their median highlighted. Statistical significance level was considered at *P* < 0.05.

## Results

### Region-specific effects of ageing on BEC features

We assessed BEC phenotype with age, in young (2–4 months) and aged (18–20 months) mice, across the prefrontal cortex, hippocampus, and corpus callosum.

**Figure 1:**
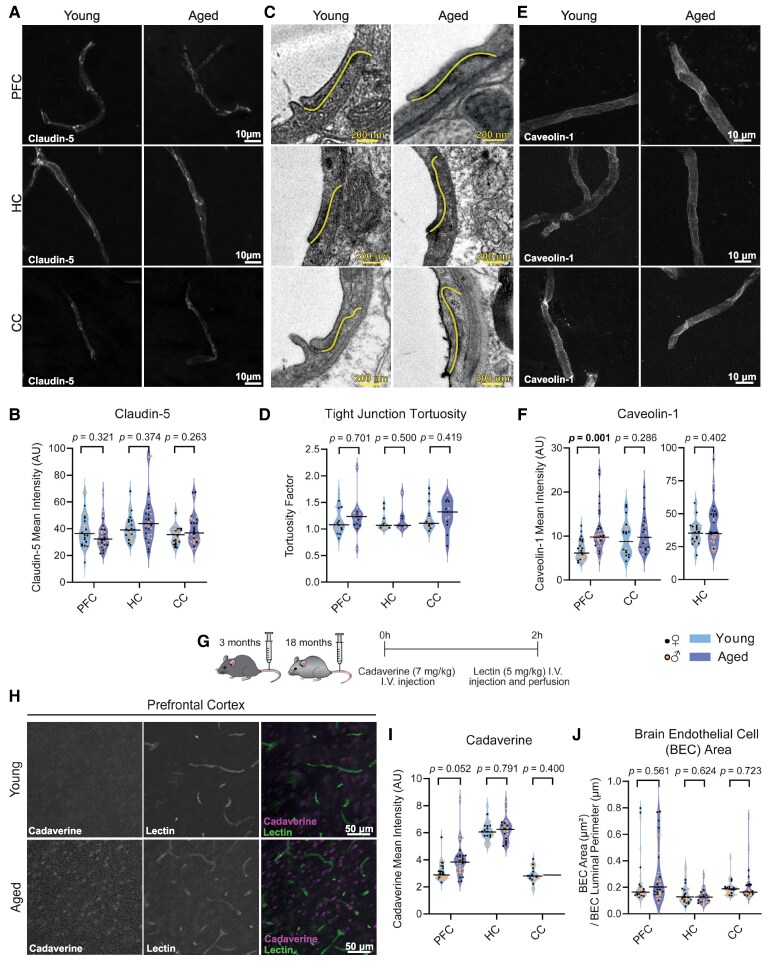
**Region-specific effects of ageing on BEC features.** Figure 1 relates to Supplementary Fig. 1. (**A**): Representative images of claudin-5 immunostaining in the PFC, HC and CC of young and aged mice. (**B**): Quantification of claudin-5 immunostaining intensity in individual vessels. PFC and HC: *n* = 32 vessels from 8 mice (4 females, 4 males). CC: *n* = 32 vessels from 8 mice (4 females, 4 males). (**C**): Representative images of PFC, HC and CC tight junctions (coloured in yellow) of young and aged mice. (**D**): Quantification of tight junction tortuosity in individual vessels in the PFC, HC and CC of young and aged mice. PFC: *n* = 15–19 vessels from 6 mice (3 females, 3 males). HC: 8–13 vessels from 5 mice (young: 3 females, 2 males; aged: 2 females, 3 males). CC: 13–20 vessels from 6 mice (3 females, 3 males). (**E**): Representative images of caveolin-1 immunostaining in the PFC, HC and CC of young and aged mice. (**F)**: Quantification of caveolin-1 immunostaining intensity in individual vessels. PFC and HC: *n* = 32 vessels from 8 mice (4 females, 4 males). CC: *n* = 24 vessels from 8 mice (4 females, 4 males). (**G**): Schematic representing the experimental design to determine the BBB extravasation of cadaverine in young and aged mice. (**H**): Representative images of cadaverine signal (magenta) in the PFC of young and aged mice. Vessels are stained with lectin (green). (**I**): Quantification of cadaverine immunostaining intensity in the PFC, HC and CC. PFC: *n* = 32 images from 8 mice (4 females, 4 males). HC: *n* = 24 images from 8 mice (4 females, 4 males). CC: *n* = 24 images from 8 mice (4 females, 4 males). (**J**): Quantification of BEC area of individual vessels in the PFC, HC, and CC of young and aged mice. PFC and CC: *n* = 30 vessels from 6 mice (3 females, 3 males). HC: 25–30 vessels from 5 to 6 mice (young: 3 females, 3 males; aged: 2 females, 3 males). See Table 2 for further description on each measurement. Data were analysed using linear mixed-effects modelling (LMM; Intensity ∼ Age + (1 | Animal) + (1 | Sex)) followed by Type 3 ANOVA with Satterthwaite approximation. The horizontal bars on the violin plots represent the median. In B, D, F, and J each datapoint represents an individual vessel; in I, each data point represents one picture. AU, arbitrary units (from 0 to 255); CC, corpus callosum; HC, hippocampus; PFC, prefrontal cortex.

As tight junctions are an essential feature of BECs, we quantified the tight junction protein claudin-5 by immunofluorescence and tight junction tortuosity^35^ with TEM in young and aged mouse brain tissue. We found no age-associated differences in these measures in any of the three brain regions of study (Fig. 1A–D and Supplementary Fig. 1A).

BECs are characteristic for their low levels of caveolae-mediated transcytosis^36^ which can be altered by age.^12^ Thus, we investigated changes in expression of caveolin-1, the principal component of caveolae,^37^ in BECs by immunofluorescence. We observed increased expression of caveolin-1 in the aged prefrontal cortex when compared with their young counterparts, with no differences in the aged hippocampus or corpus callosum (Fig. 1E, F). To assess if the age-dependent increase of caveolin-1 in the prefrontal cortex was driven by a specific sex, we included sex as a fixed effect in our statistical model (Supplementary Table 1). Interestingly, we observed that caveolin-1 expression was statistically higher in females than males in all three brain regions, independently of age (Supplementary Fig. 1B–D, Supplementary Table 1). In addition, while there was no significant interaction between age and sex in the prefrontal cortex, i.e. caveolin-1 increased in both aged females and males (Supplementary Fig. 1B, Supplementary Table 1), we detected an age × sex interaction effect for the hippocampus, in which caveolin-1 appears increased in aged females and subtly decreased in aged males (Supplementary Fig. 1C, Supplementary Table 1). Incidentally, no sex-associated differences were observed for claudin-5 and tight junction tortuosity (Supplementary Table 1). Nonetheless, we advise age × sex comparisons should be taken with caution due to the reduction of statistical power that derives from making comparisons between smaller groups. Transcellular transport enhancement can lead to BBB permeability increase,^12,38^ so we next evaluated potential permeability changes associated to caveolin-1 upregulation by quantifying the extravasation of a small dye that does not cross the BBB under healthy conditions (cadaverine-546 of 800 Da) (Fig. 1G). We observed a subtle increase (*P* = 0.052) of cadaverine transport across the BBB in the prefrontal cortex of aged compared with young mice, not apparent in hippocampus or corpus callosum (Fig. 1H, I and Supplementary Fig. 1E, F), and not influenced by sex (Supplementary Table 1). There was no age-related BEC size alteration observed with TEM in any of the three brain regions of interest (Fig. 1J and Supplementary Figs 2–4, Supplementary Table 1).

Our data suggests that with age there is increased caveolin-1 mediated transcellular permeability in the female and male prefrontal cortex and female hippocampus, with no changes in the corpus callosum. Meanwhile, other BEC features like tight junctions or size remain spared.

### Basement membrane age-associated alterations

**Figure 2:**
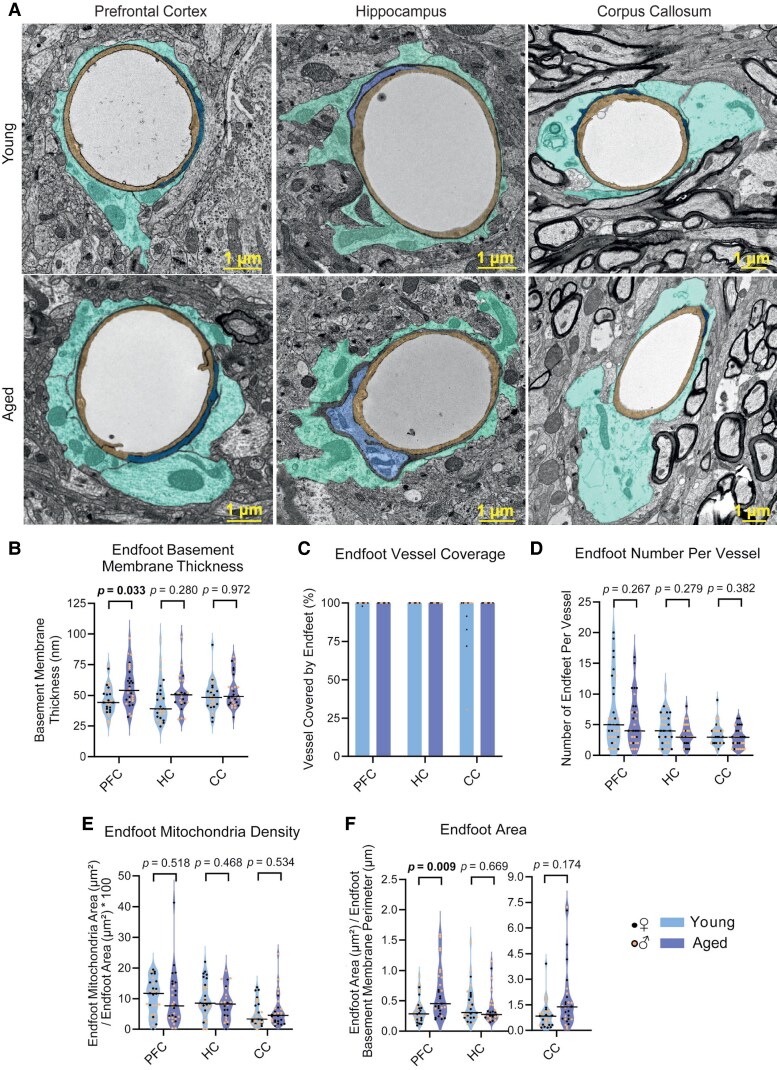
**Region-specific effects of ageing in astrocyte endfoot ultrastructural features.** Figure 2 relates to Supplementary Fig. 6. (**A**): Representative images of blood vessels in the PFC, HC and CC of young and aged mice. BECs are coloured in yellow, pericytes are coloured in blue and endfeet are coloured in cyan. (**B–F**): Quantification of endfoot basement membrane thickness (**B**), endfoot vessel coverage (**C**), endfoot number per vessel (**D**), endfoot mitochondria density (**E**), and endfoot area (**F**) in individual vessels in the PFC, HC and CC of young and aged mice. See Table 2 for further description on each measurement. PFC and CC: *n* = 30 vessels from 6 mice (3 females, 3 males). HC: 25–30 vessels from 5–6 mice (young: 3 females, 3 males; aged: 2 females, 3 males). Data were analysed using LMM [Intensity ∼ Age + (1 | Animal) + (1 | Sex)] followed by Type 3 ANOVA with Satterthwaite approximation. The horizontal bars on the violin plots represent the median. Each datapoint represents an individual vessel. Figure 2C does not display statistical analysis because, for most of the conditions, astrocyte coverage was 100% in all vessels and this led to lack of variance in the dataset which made it unsuitable for statistical analysis. In this figure, some points in young CC have a value <100%; this was due to the presence of ambiguous dark endfoot-like structures that were not included in the counting. Only clear endfeet were considered because we could not determine the cell identity of the dark structures. CC, corpus callosum; HC, hippocampus; PFC, prefrontal cortex.

Using TEM, we also assessed basement membrane thickness, which plays a critical role as an interface for cell-cell communication and which has been previously shown to increase with age.^5-7,9^ Unlike previous studies, in our analysis we distinguished the basement membrane segments located between BECs and pericytes from those between BECs and astrocyte endfeet to assess if they were differentially altered. Consistent with earlier findings, we observed an increase in basement membrane thickness with age, but only in the prefrontal cortex and only for the basement membrane between astrocyte endfeet and BECs (Fig. 2A, B, Supplementary Figs. 2–4 and 5A). We found no statistically significant interaction between age and sex, suggesting that both female and male samples contributed to the basement membrane increase observed in the prefrontal cortex (Supplementary Fig. 6A, Supplementary Table 1). This result, together with the increase in caveolin-1 expression (Fig. 1E, F), indicated higher BBB susceptibility to early ageing in the prefrontal cortex when compared with hippocampus and corpus callosum, and it prompted us to investigate if these alterations may coexist with changes in astrocyte endfeet.

### Region-specific effects of ageing in astrocyte endfeet

We began by evaluating molecular changes in astrocyte endfeet by assessing the expression levels of well-known endfoot proteins: AQP-4, β-dystroglycan, and dystrophin by immunofluorescence. AQP-4 is a water-selective channel with proposed roles in brain water homeostasis and glymphatic flow.^39,40^ We calculated the polarization ratio of vascular versus parenchymal AQP-4 expression in prefrontal cortex, hippocampus, and corpus callosum and identified no age-associated differences (Supplementary Fig. 7A–C, H–J, and O–Q). We also examined β-dystroglycan and dystrophin, two components of the dystrophin-associated protein complex that anchors AQP-4 to the plasma membrane of the astrocyte endfoot.^19,41^ Although there was no change in these proteins with age in the prefrontal cortex or corpus callosum (Supplementary Fig. 7D–G and R–U), hippocampal β-dystroglycan was significantly higher in aged vessels when compared with the young ones (Supplementary Fig. 7K–N).

We next used TEM to investigate other, structural characteristic features of astrocyte endfeet. Endfoot coverage and the number of endfeet surrounding each vessel, as well as mitochondrial density displayed no differences with age across any of the three brain regions of study (Fig. 2C–E and Supplementary Figs 2–4). Despite previous reports showing that pericyte vessel-coverage is reduced with age using immunofluorescence (in 20–24 months old mice),^12,30,31^ we observed no alterations of pericyte coverage in our age range (18–20 months) using TEM (Supplementary Fig. 5B, C) or CD13 immunostaining in any of the brain regions analysed (Supplementary Fig. 5D–G). However, astrocyte endfeet were significantly hypertrophic in the prefrontal cortex of aged mice compared with young ones, with no differences in hippocampus or corpus callosum (Fig. 2F and Supplementary Figs 2–4). Akin to caveolin-1 and the endfoot basement membrane, no statistically significant interaction was found between age and sex for prefrontal cortex endfoot size, although endfeet appeared bigger overall in males (Supplementary Fig. 6B, Supplementary Table 1).

In summary, we observed that at early stages of ageing, in 18–20-month-old mice, changes to the BBB are subtle, with most of the BBB age-dependent changes presenting in the prefrontal cortex (Table 3).

**Table 3:** Compilation of *P* values obtained from all the statistical comparisons between aged and young animals in this manuscript, using LMM [measurement ∼ age + (1 | animal) + (1 | sex)] followed by type 3 ANOVA with satterthwaite approximation.

Parameter	Prefrontal cortex	Hippocampus	Corpus callosum
Claudin-5	0.321	0.374	0.263
Tight junction tortuosity	0.701	0.500	0.419
Caveolin-1	**0**.**002**	0.305	0.269
Cadaverine permeability	0.052	0.791	0.400
BEC area	0.561	0.624	0.723
Pericyte basement membrane	0.075	0.736	0.060
Endfoot basement membrane	**0**.**033**	0.280	0.972
Aquaporin-4 vascular	0.675	0.825	0.148
Aquaporin-4 parenchymal	0.947	0.902	0.434
Aquaporin-4 vascular/parenchymal	0.791	0.901	0.966
β-Dystroglycan	0.084	**0**.**006**	0.735
Dystrophin	0.459	0.890	0.971
Endfoot coverage	N/A	N/A	N/A
Endfoot number	0.267	0.279	0.382
Endfoot mitochondrial density	0.518	0.468	0.534
Pericyte vessel coverage	0.201	0.718	0.554
Pericyte area	0.351	0.760	0.578
Endfoot area	**0**.**009**	0.669	0.174

Statistically significant results of *P* < 0.05 are highlighted in bold.

## Discussion

Here we examine BBB age-related alterations between three brain regions that are highly relevant to cognition: the prefrontal cortex, hippocampus, and the corpus callosum. We discovered that the most vulnerable area to early BBB ageing is the prefrontal cortex with increased caveolae-mediated transcytosis, basement membrane thickness, and endfoot size (Table 3). This was followed by the hippocampal BBB that presented a sex-dependent alteration of caveolin-1 expression and higher expression of β-dystroglycan in astrocyte endfeet with age (Table 3 and Supplementary Table 1), and surprisingly, despite the high sensitivity of white matter to age-associated cerebral small vessel disease,^42^ we observed no differences in the corpus callosum BBB (Table 3).

It has been suggested that BEC alterations with age lead to failure in both paracellular^7,8,43^ and transcellular^12^ barrier mechanisms, with the observation of increased age-associated BBB permeability in both mouse and human in some studies,^12,13,31,43-45^ but not in others.^8,46^ Whilst altered expression or re-arrangement of tight junction proteins like claudin-5 and ZO-1 have been reported in the brains of aged mice and humans with immunostaining and TEM,^7,8,43^ our study found neither. This discrepancy may be due to differences in the regions of study, ages, and the techniques used. For example, Frias-Anaya *et al*.^7^ observed that 3D-TEM is needed to be able to detect alterations of the tight junction structure with age in 24-month-old mice,^7^ and only observed alterations with age in the cortex and not the hippocampus. We observed a subtle increase in BBB permeability in the prefrontal cortex (Fig. 1G–I) and increased expression of caveolin-1 with age (Fig. 1E, F), which was consistent with previously described age-associated increased caveolae-mediated transcytosis.^12^ While our study focused on the prefrontal cortex, previous research on BBB permeability suggests that other cortical regions may exhibit higher susceptibility to aging, as observed in vivo in both humans^14,47^ and rodents.^48^ In the future, it will be interesting to compare how these macroscopic observations translate at the microscopic scale. Moreover, several studies have used tracer-based approaches to investigate sex-differences in BBB permeability. The evidence so far shows a higher age-related BBB vulnerability in males compared with females, especially in cortical areas, observed in both humans^14,47,49^ and rodents.^48^ Although our data show higher caveolin expression in the prefrontal cortex and hippocampus of females compared with males, we did not observe any sex-dependent differences in BBB permeability to cadaverine with age. The discrepancy between our results and previous research may be attributable to the limited number of biological replicates used in our age-by-sex interaction analyses, and it could also reflect the use of different methodologies to assess BBB function.

Increased basement membrane area in the rodent cortex, hippocampus, thalamus, and striatum has been associated with age in numerous TEM studies.^5-7,9^ In our analysis, we distinguished between the pericyte and endfoot basement membrane and found the endfoot basement membrane was significantly thicker in the 18–20 month aged prefrontal cortex (Fig. 2B) but not in hippocampus or corpus callosum. Why the basement membrane increases in size with age is not known; but previous studies have shown alterations in its protein composition in cortex and hippocampus with age.^9,45,50^

In line with an increased BBB susceptibility to ageing in the prefrontal cortex, we identified larger astrocyte endfeet in this region in aged mice when compared with the young counterparts, again not observed in hippocampus or corpus callosum endfeet. Hypertrophic endfeet have previously been observed in the striatum of aged rats.^5^ A possibility is that these endfeet are filled with water, although appearance of their cytoplasm was very similar to the one of young endfeet (Supplementary Fig. 2). Whilst some studies have reported altered age-associated AQP-4 expression in cortical astrocytes,^11,51,52^ we found no AQP-4 expression differences in any region (Supplementary Fig. 7). AQP-4 function can be regulated by its removal from the plasma membrane without the need of relocating within the cell,^53^ a mechanism which would not be detected by our quantification method. Additionally, higher resolution endfoot TEM images could help determine the composition of hypertrophic endfeet, to investigate alternative explanations to an increase in the water content. Interestingly, despite the endfoot change in morphology, neither the number of endfeet nor their vessel coverage was altered. Indeed, virtually complete endfoot coverage of the blood vessels seems to be a very robust feature of the BBB.^15,54^ Mills and colleagues demonstrated that when one endfoot is removed through two-photon ablation, neighbouring astrocytes rapidly extend their processes to replace the ablated structure within minutes even in older mice.^55^

This study has some limitations. It is possible that the BBB is altered in ways that our experimental design does not detect, and that although they might be subtle could have important impact on the physiology of the BBB. In addition, while we see region-dependent multicellular alterations with age, we do not know if there is a cause-effect among them or if they just represent independent coexisting events. Similarly, we are unclear whether age-driven BBB differences between brain regions relate to parenchymal alterations or are intrinsic to the vasculature. Many studies identified the cerebral cortex as particularly vulnerable to ageing both structurally and functionally^56^ and, like with the vasculature,^14,47,48^ age affects the parenchyma in an area-specific manner.^57^ For instance, the prefrontal cortex shows one of the biggest volume losses with advanced age,^58,59^ while functional connectivity studies identified parietal, cingulate, and occipital cortices as the regions more susceptible to age,^60,61^ coinciding with the cortical areas in which the vasculature is particularly affected. More comprehensive parallel proteomics and functional studies will give us a more precise picture of the altered pathways that may lead to BBB dysfunction with age. In addition, it would be interesting to include subcortical brain regions that are particularly susceptible to age-related human vascular disease.^42^

In conclusion, previous research has shown diverse BBB dysfunction phenotypes associated with age in mouse and human. However, the confined focus of each study on either specific brain regions, stages of ageing, or phenotypes has made it difficult to determine if BBB susceptibility in ageing is region-dependent and whether BBB cell-specific alterations correlate with each other. Here, by systematically comparing the phenotypes of the different capillary-BBB components across three brain regions, we demonstrate region-specific changes of the BBB with age. In our study, the hippocampus and corpus callosum appeared resilient to early ageing, while the prefrontal cortex was the most affected region with increased caveolae-mediated transcytosis, basement membrane thickness, and astrocyte endfoot size. New studies will be needed to better understand the mechanisms that lead to region-specific early events of BBB dysfunction, how they increase susceptibility to disease, and if their prevention could delay pathology.

## Supplementary Material

fcaf332_Supplementary_Data

## Data Availability

TEM images and masks for analyses are accessible at Zenodo: I.B.-F., K.G.-B., H.J.V., and B.D.C. (2025). Multiregional BBB phenotyping identifies the prefrontal cortex as the most vulnerable region to ageing in mice—https://zenodo.org/records/14900870. The code used for liner mixed-effect model analyses in this manuscript is available at https://github.com/Diaz-Castro-Lab/Regional-blood-brain-barrier-ageing-LMM-script.
