# Supplementary material for: Multiregional blood-brain barrier phenotyping identifies the prefrontal cortex as the most vulnerable region to ageing in mice
Source: Brain Commun. 2025 Sep 10;7(5):fcaf332. doi: 10.1093/braincomms/fcaf332 (PMC12455408; doi:10.1093/braincomms/fcaf332)
Supplement: fcaf332_Supplementary_Data [file fcaf332_supplementary_data.zip › Supplementary_Figures.pdf]

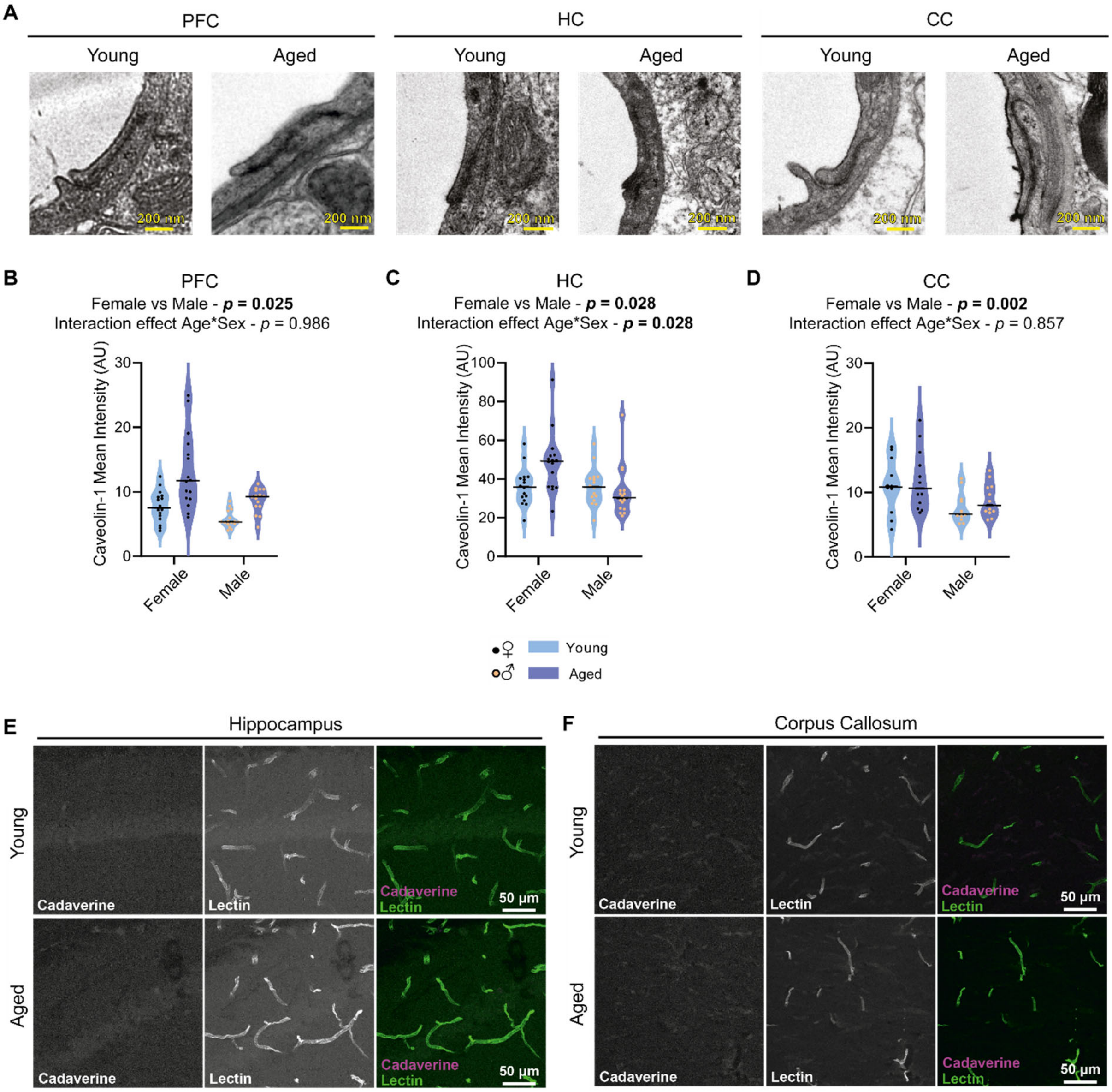

Supplementary Figure 1

Supplementary Figure 1. A: Representative images shown in Fig. 1C, here without the yellow line highlighting the tight junctions. B-C: Quantification of caveolin-1 immunostaining intensity in individual vessels shown in Fig. 1F, here separating the female and male groups for PFC (B), HC (C) and CC (D). PFC and HC: n=16 vessels per age/sex, from 8 mice (4 females, 4 males). CC: n=12 vessels per age/sex, from 8 mice (4 females, 4 males). Data were analysed considering both age and sex as fixed effects using linear mixed-effect modelling (LMM; Measurement ~ Age * Sex + (1|Animal)) followed by Type 3 analysis of variance (ANOVA) with Satterthwaite approximation. E-F: Representative images of cadaverine signal (magenta) with vessels stained with lectin (green) in hippocampus (E) and corpus callosum (F). Abbreviations: PFC: prefrontal cortex, HC: hippocampus, CC: corpus callosum.

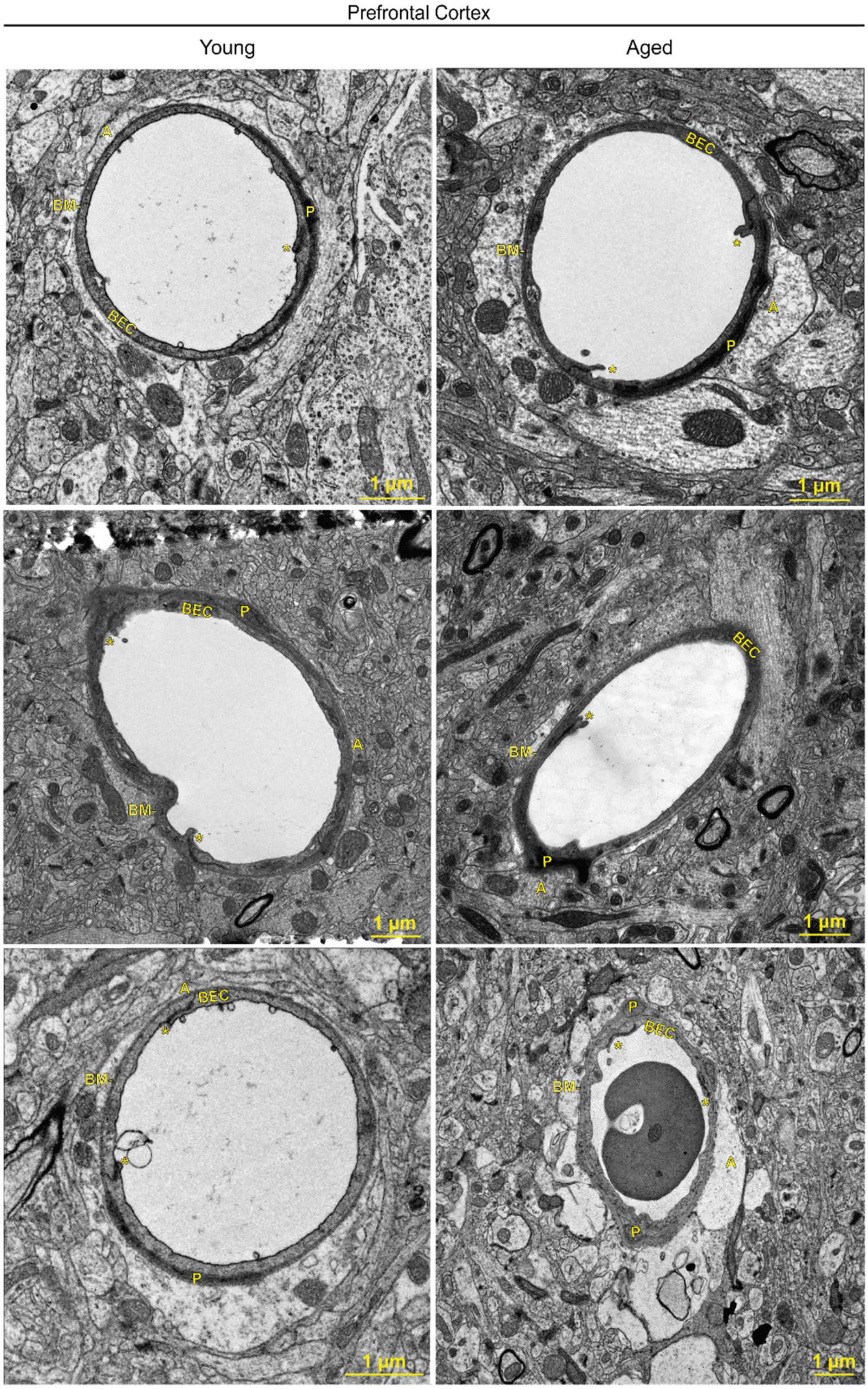

Supplementary Figure 2

Supplementary Figure 2. Representative images of blood vessels in the prefrontal cortex of young and aged mice. BEC: brain endothelial cell, *: tight junction, P: pericyte, BM: basement membrane, A: astrocyte.

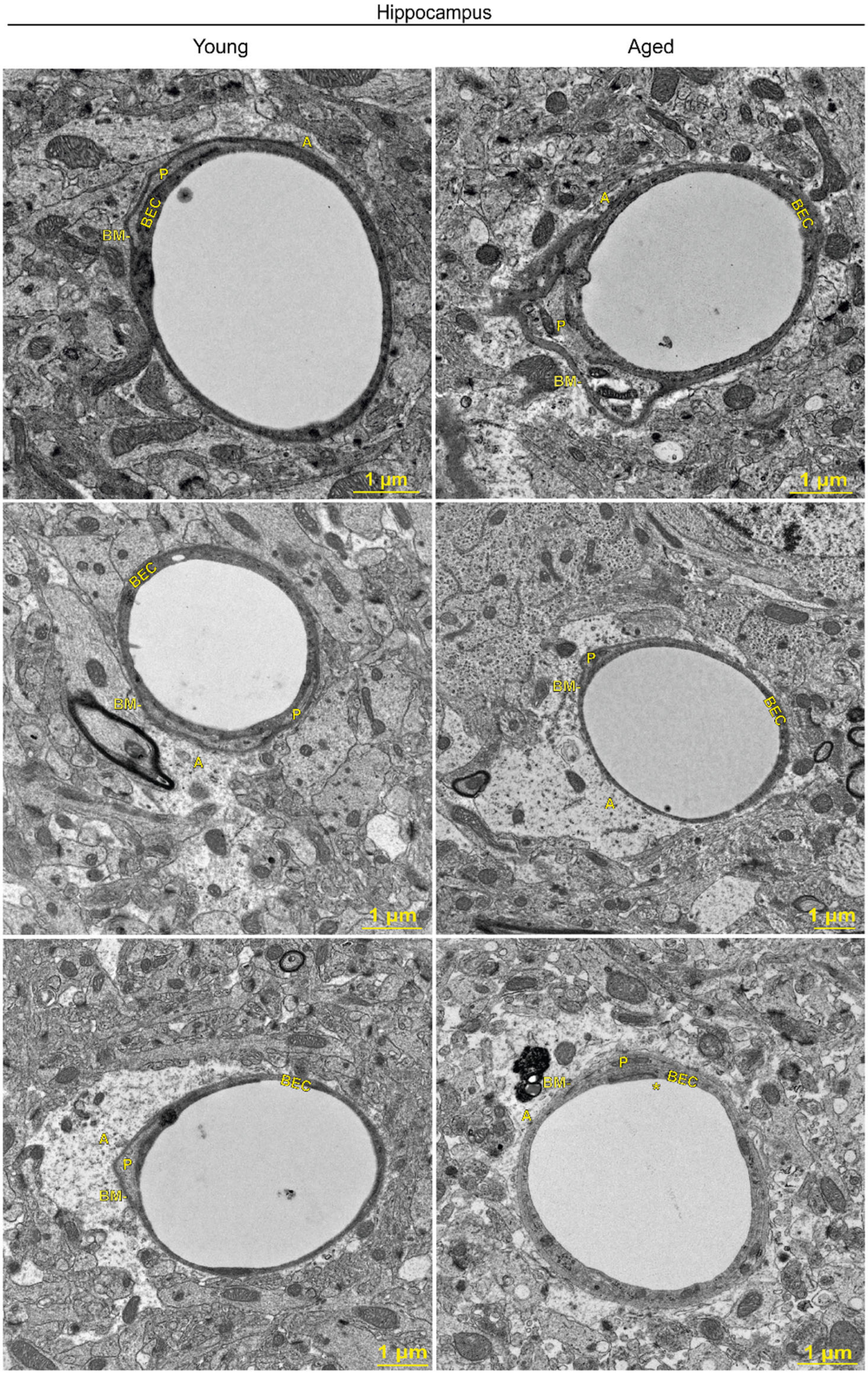

Supplementary Figure 3

Supplementary Figure 3. Representative images of blood vessels in the hippocampus of young and aged mice. BEC: brain endothelial cell, *: tight junction, P: pericyte, BM: basement membrane, A: astrocyte.

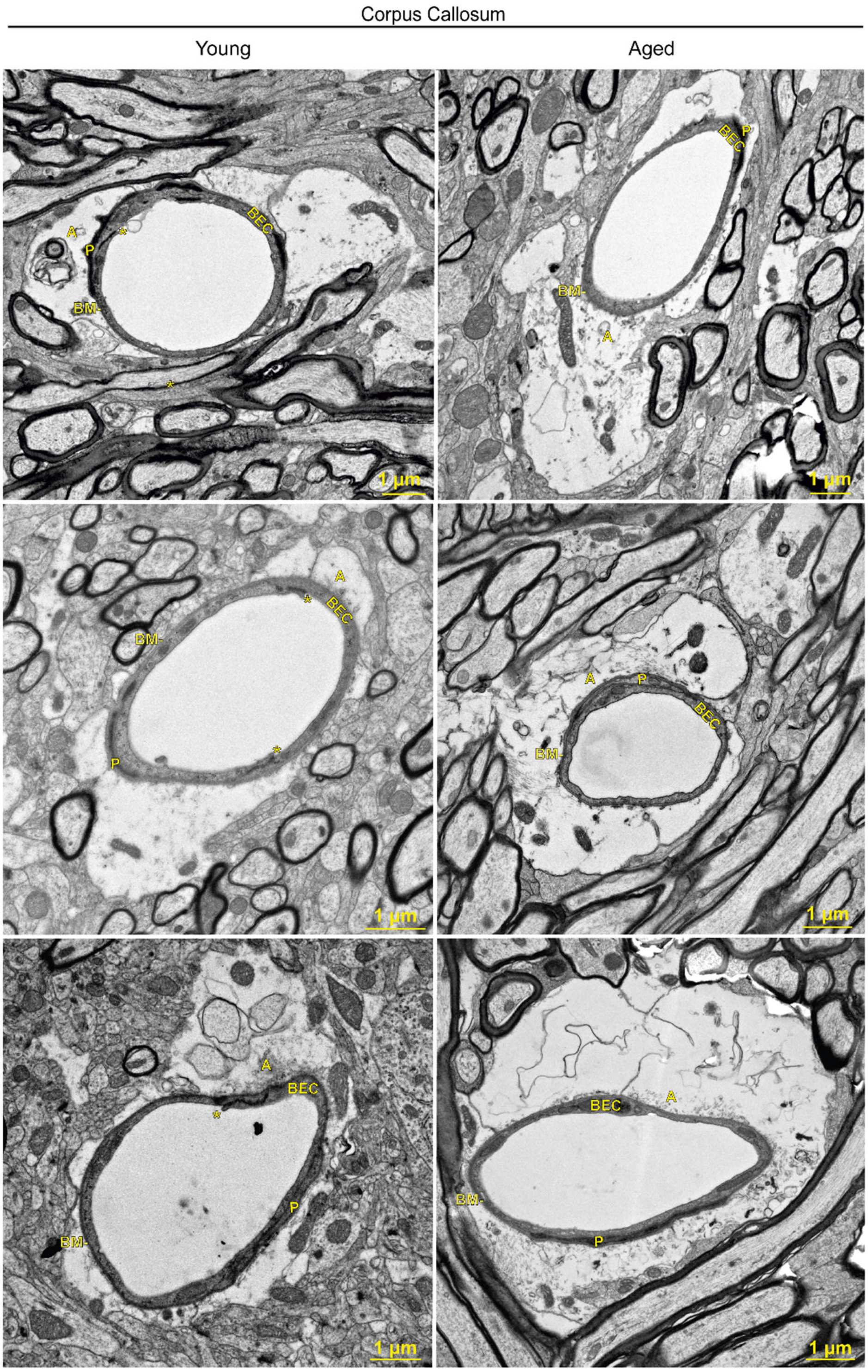

Supplementary Figure 4

Supplementary Figure 4. Representative images of blood vessels in the corpus callosum of young and aged mice. BEC: brain endothelial cell, *: tight junction, P: pericyte, BM: basement membrane, A: astrocyte.

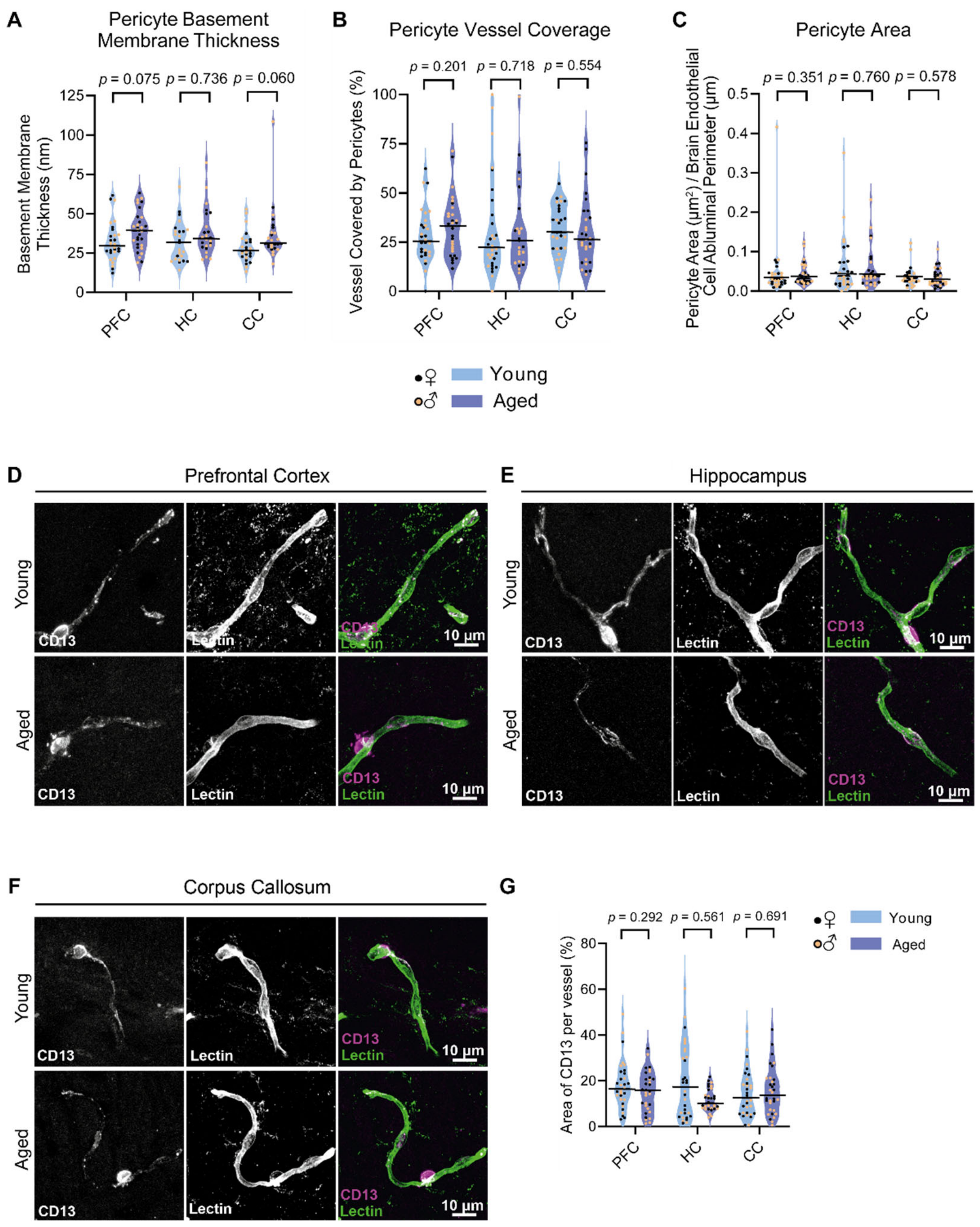

Supplementary Figure 5

Supplementary Figure 5. A-C: Quantification of pericyte basement membrane thickness (A), pericyte vessel coverage (B), and pericyte area (C) in individual vessels in the PFC, HC, and CC of young and aged mice. See Table 3 for further description on each measurement. PFC and CC: n=30 vessels from 6 mice (3 females, 3 males). HC: 25-30 vessels from 5-6 mice (young: 3 females, 3 males; aged: 2 females, 3 males). D-F: Representative images of CD13 immunostaining in the PFC (D), HC (E), and CC (F) of young and aged mice. G: Quantification of percent area of the blood vessel (labelled with lectin in green) that colocalizes with the CD13 signal (magenta). PFC, HC and CC: n=32 vessels from 8 mice (4 females, 4 males). Data were analysed using LMM (Intensity ~ Age + (1 | Animal) + (1 | Sex)) followed by Type 3 analysis of variance (ANOVA) with Satterthwaite approximation. The horizontal bars on the violin plots represent the median. Each datapoint represents an individual vessel. Abbreviations: PFC: prefrontal cortex, HC: hippocampus, CC: corpus callosum.

Prefrontal Cortex

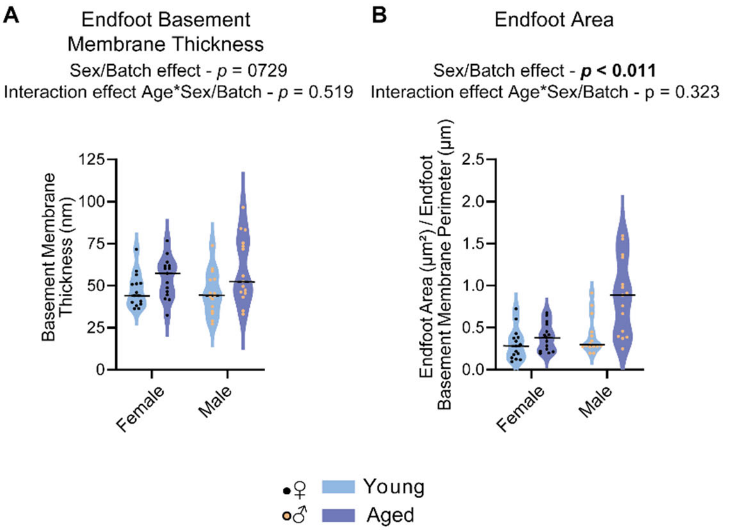

Supplementary Figure 6

Supplementary Figure 6. A: Quantification of endfoot basement membrane thickness in individual vessels shown in Fig. 2B, here separating the female and male groups for prefrontal cortex. n=15 vessels per age/sex from 6 mice (3 females, 3 males). B: Quantification of endfoot area in individual vessels shown in Fig. 2F, here separating the female and male groups for prefrontal cortex. n=15 vessels per age/sex from 6 mice (3 females, 3 males). Data were analysed considering both age and sex as fixed effects using linear mixed-effect modelling (LMM; Measurement ~ Age * Sex/Batch + (1|Animal)) followed by Type 3 analysis of variance (ANOVA) with Satterthwaite approximation. In the electron microscopy experiments, female and male samples were processed at separately; thus, sex and batch effects cannot be statistically distinguished. Each datapoint represents an individual vessel.

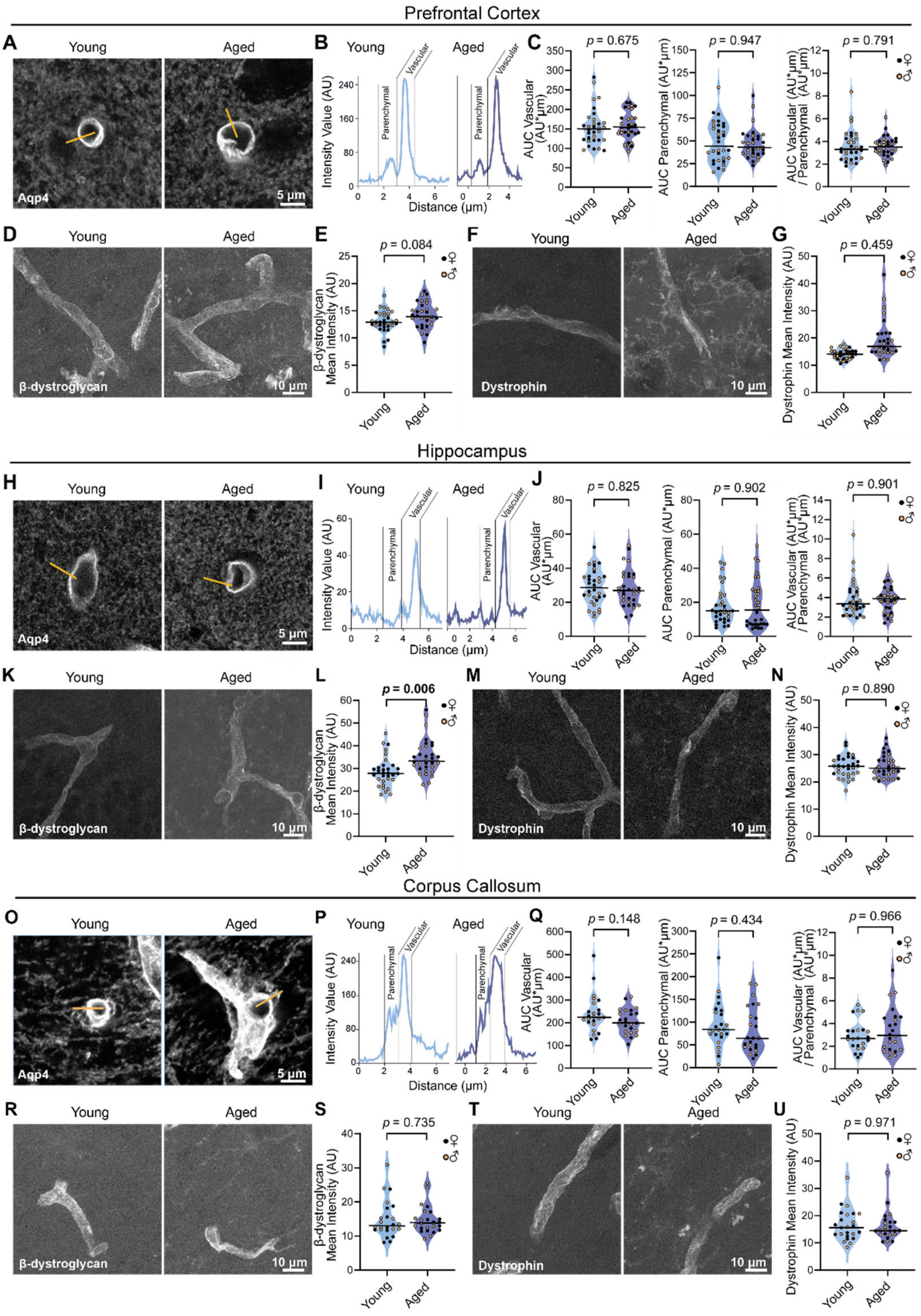

Supplementary Figure 7

Supplementary Figure 7. Region-specific effects of ageing in astrocyte endfoot protein expression. A-B: Representative images of AQP-4 immunostaining in the PFC of young and aged mice. For the analysis, a yellow line was drawn transversally to individual vessels and the intensity profile along the line was plotted (B), delineating AQP-4 signal across vascular and parenchymal regions. C: Quantification of the AUC in the vascular (left) and parenchymal (middle) regions and the vascular-to-parenchymal AUC ratio (right) in individual vessels of the PFC. D: Representative images of ß-dystroglycan immunostaining in the PFC of young and aged mice. E: Quantification of ß-dystroglycan staining intensity in individual vessels of the PFC. F: Representative images of dystrophin immunostaining in the PFC of young and aged mice. G: Quantification of dystrophin staining intensity in individual vessels of the PFC. H: Representative images of AQP-4 immunostaining in the HC of young and aged mice. I: Intensity profiles corresponding to examples shown in H. J: Quantification of the AUC in the vascular (left) and parenchymal (middle) regions and the vascular-to-parenchymal AUC ratio (right) in individual vessels of the HC. K: Representative images of ß-dystroglycan immunostaining in the HC of young and aged mice. L: Quantification of ß-dystroglycan staining intensity in individual vessels of the HC. M: Representative images of dystrophin immunostaining in the HC of young and aged mice. N: Quantification of dystrophin staining intensity in individual vessels of the HC. O: Representative images of AQP-4 immunostaining in the CC of young and aged mice. P: Intensity profiles corresponding to examples shown in O. Q: Quantification of the AUC in the vascular (left) and parenchymal (middle) regions and the vascular-to-parenchymal AUC ratio (right) in individual vessels of the CC. R: Representative images of ß-dystroglycan immunostaining in the CC of young and aged mice. S: Quantification of ß-dystroglycan staining intensity in individual vessels of the CC. T: Representative images of dystrophin immunostaining in the CC of young and aged mice. U: Quantification of dystrophin staining intensity in individual vessels of the CC. PFC and HC: n=32 vessels from 8 mice (4 females, 4 males). CC: n=24 vessels from 8 mice (4 females, 4 males). Data were analysed using LMM (Measurement ~ Age + (1 | Animal) + (1 | Sex)) followed by Type 3 analysis of variance (ANOVA) with Satterthwaite approximation. The horizontal bars on the violin plots represent the median. Each datapoint represents an individual vessel. Abbreviations: AQP-4: aquaporin 4, AU: arbitrary units (from 0 to 255), AUC: area under the curve, PFC: prefrontal cortex, HC: hippocampus, CC: corpus callosum.
